# Hearing impairment increases the risk of distal radius, hip, and spine fractures: A longitudinal follow-up study using a national sample cohort

**DOI:** 10.1371/journal.pone.0192820

**Published:** 2018-02-13

**Authors:** So Young Kim, Joon Kyu Lee, Songyong Sim, Hyo Geun Choi

**Affiliations:** 1 Department of Otorhinolaryngology-Head & Neck Surgery, CHA Bundang Medical Center, CHA University, Seongnam, Korea; 2 Department of Orthopaedic Surgery, Hallym University College of Medicine, Anyang, Korea; 3 Department of Statistics, Hallym University, Chuncheon, Korea; 4 Department of Otorhinolaryngology-Head & Neck Surgery, Hallym University College of Medicine, Anyang, Korea; Medical College of Wisconsin, UNITED STATES

## Abstract

**Objective:**

Hearing impairment has been suggested to increase the risk of falls. However, most previous studies were conducted in an older population without classification of the fracture regions. This study aimed to delineate the risk of each fracture type in all age populations.

**Methods:**

The Korean National Health Insurance Service-National Sample Cohort was collected from 2002 to 2013. A total of 4,854 severe hearing-impaired and 1,354 profound hearing-impaired participants were matched for age, group, sex, income group, and region of residence with 19,416 and 5,416 control participants, respectively. The fracture diagnosis was based on the International Classification of Disease (ICD-10) codes as follows: distal radius fracture (S525), hip fracture (S720, S721, S722), and spine fracture (S220, S32). Crude (simple) and adjusted hazard ratios (HRs) for each fracture associated with severe or profound hearing impairment were analyzed using the Cox proportional hazard model.

**Results:**

The severe hearing-impaired group had an increased risk of distal radius fracture, hip fracture, and spine fracture compared with the control group (adjusted HR = 1.67, 95% CI = 1.38–2.03, P < 0.001 for hip fracture). The profound hearing-impaired group had an increased risk of hip and spine fracture (adjusted HR = 2.21, 95% CI = 1.44–3.39, P < 0.001 for hip fracture).

**Conclusion:**

The risk of distal radius fracture, hip fracture, and spine fracture was increased in the severe hearing-impaired group compared with the control group.

## Introduction

Fractures are prevalent injuries that occur across all age groups. For example, the incidence of distal radius fracture was estimated to be approximately 278 per 100,000 people and 709 per 100,000 women in Europe [1,2]. The incidence of distal radius fracture was suggested to have increased consistently in recent years [1,3]. This elevated incidence of fractures may originate from individuals working longer and the growing elderly proportion of the population [1]. Fractures impair physical activities in daily living and in the workplace. Thus, the socioeconomic burden of fractures is very high. It was reported that the medical costs for fractures were more than 1 billion Euros in 2002 in Italy [4]. In addition, fractures increase the need for hospital care. Approximately 93.0% of hip fracture patients are reported to have required hospitalization [5].

Various factors increase the risk of fractures according to types of fractures including age and osteoporosis, the latter of which is a leading cause of fracture [6]. More than 8.9 million osteoporotic fractures occur annually worldwide [7]. Osteoporotic fractures account for approximately 81% of all fractures in female individuals who are ≥50 years old [8]. Osteoporotic fractures present in various regions, including the hip and several skeletal regions, such as the forearm, humerus, ankle, and vertebrae [5]. Among these regions, the most common osteoporotic fractures were hip (18%), forearm (16%), and vertebral (15%) fractures [7]. However, osteoporosis is often undiagnosed or untreated and can even be detected after an osteoporotic fracture [9,10]. Falls and physical trauma are other main causes of fracture in younger populations [11].

Hearing impairment has been reported to increase the risk of falls, although there have been conflicting reports in the literature. A meta-analysis demonstrated a 1.72 pooled odds ratio of falls in hearing-impaired elderly individuals (95% CI = 1.07–2.37) [12]. However, few studies have investigated the risk of falls or fractures across all age populations and instead limited the studies to an elderly population. Additionally, the fracture region was either not specified or was confined to just one affected fracture site.

The hypothesis of this study was that hearing impairment may elevate the risk of fractures in all age groups besides the elderly population. In addition, severe hearing impairment may impact fracture risk irrespective of the fracture site. To evaluate these hypotheses, we conducted a longitudinal follow-up study on the risk of fracture, including distal radius, hip, and spine fractures, in severely or profoundly hearing-impaired populations across the lifespan.

## Materials and methods

### Study population and data collection

The ethics committee of Hallym University (2014-I148) approved the use of these data. Written informed consent was exempted by the Institutional Review Board.

This national cohort study relied on data from the Korean National Health Insurance Service-National Sample Cohort (NHIS-NSC). The Korean National Health Insurance Service (NHIS) selects samples directly from the entire population database to prevent non-sampling errors. Approximately 2% of the samples (one million) were selected from among the entire Korean population (50 million). These selected data can be classified at 1,476 levels (age [18 categories], sex [2 categories], and income level [41 categories]) using randomized stratified systematic sampling methods via proportional allocation to represent the entire population. After data selection, the appropriateness of the sample was verified by a previous study [13]. The details of the methods used to perform these procedures are provided by the National Health Insurance Sharing Service [14]. This cohort database included (i) personal information, (ii) health insurance claim codes (procedures and prescriptions), (iii) diagnostic codes according to the International Classification of Disease-10 (ICD-10), (iv) death records from the Korean National Statistical Office (using the Korean Standard Classification of disease), (v) socioeconomic data (residence and income), and (vi) medical examination data for each participant over a period ranging from 2002 to 2013.

Because all Korean citizens are recognized by a 13-digit resident registration number from birth to death, exact population statistics can be determined using this database. It is mandatory for all Koreans to enroll in the NHIS. All Korean hospitals and clinics use 13-digit resident registration numbers to register individual patients in the medical insurance system. Therefore, the risk of overlapping medical records is minimal, even if a patient moves from one place to another.

### Participant selection

Of the 1,025,340 cases with 114,369,638 medical claim codes, we included participants who were registered as hearing-impaired individuals by the Ministry of Health and Welfare. Of these participants, those who were registered with other disabilities (physical disability, brain lesion disorder, visual loss, mental retardation, psychiatric disorder, kidney disorder, and others) in the Ministry of Health and Welfare were excluded. They were divided into two groups based on the grade of impairment: severe hearing impairment (hearing threshold ≥ 60 dB in both ears; ≥ 80 dB in one ear and ≥ 40 dB in one ear) and profound hearing impairment (hearing threshold ≥ 90 dB in both ears). In Korea, to be registered as a hearing-impaired person, an individual must be evaluated three times by a pure tone audiometry test (PTA) and once by an auditory brainstem response test. The average hearing threshold of the PTA was calculated as follows: (500 Hz + 2*1000 Hz +2* 2000 Hz + 4000 Hz)/6.

**Fig 1:**
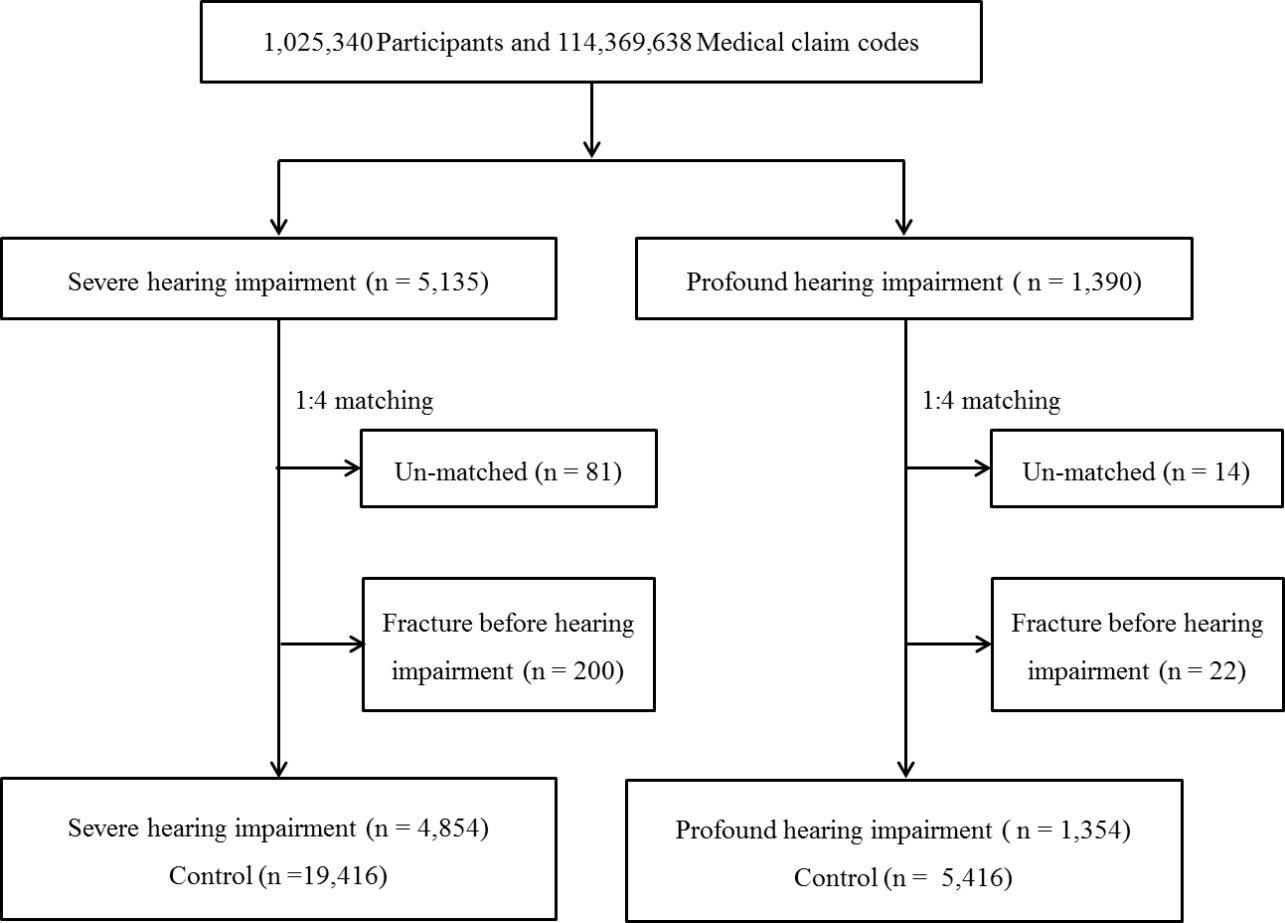
A schematic illustration of the participant selection process used in the present study. Out of 1,025,340 participants, 5,135 severe hearing-impaired and 1,390 profound hearing-impaired participants were selected. Hearing-impaired participants were matched 1:4 with a control group that did not have a hearing impairment diagnosis. Finally, 4,854 severe hearing impairment and 19,416 control participants were included as well as 1,354 profound hearing-impaired and 5,416 control participants.

The hearing-impaired participants were matched 1:4 with the participants (control group) who had never been diagnosed with a hearing impairment or with other disabilities among this cohort from 2002 through 2013. The matching was performed according to age group, sex, income group, and region of residence. To prevent selection bias when selecting the matched participants, the control group participants were sorted using a random number order and were then selected from top to bottom. It was assumed that the matched control participants were assessed at the same time as each matched hearing-impaired participant (index date). Therefore, the control group participants who died before the evaluation period of the matched hearing impairment participant were excluded. The hearing-impaired participants for whom we could not identify enough matching participants were excluded (severe hearing impairment, n = 81; profound hearing impairment, n = 14). Among them, the participants who had a history of fracture (distal radius, hip, lumbar or thoracic spine fracture) before the index date were excluded (severe hearing impairment, n = 200; profound hearing impairment, n = 22). Finally, 1:4 matching resulted in the inclusion of 4,854 severe hearing-impaired participants and 19,416 control participants as well as 1,354 profound hearing-impaired participants and 5,416 control participants (Fig 1).

### Variables

The age groups were classified using 5-year intervals: 0–4, 5–9, 10–14…, and 85+ years old. A total of 18 age groups were defined. The income groups were initially divided into 41 classes (one health aid class, 20 self-employment health insurance classes, and 20 employment health insurance classes). These groups were re-categorized into 11 classes (class 1 [lowest income]-11 [highest income]). Region of residence was divided into 16 areas according to administrative district. These regions were regrouped into urban (Seoul, Busan, Daegu, Incheon, Gwangju, Daejeon, and Ulsan) and rural (Gyeonggi, Gangwon, Chungcheongbuk, Chungcheongnam, Jeollabuk, Jeollanam, Gyeongsangbuk, Gyeongsangnam, and Jeju) areas.

Fracture history was evaluated using ICD-10 codes. Distal radius fracture was selected as Fracture of lower end of radius (S525). Hip fracture was chosen as Fracture of head and neck of femur (S720), Pertrochanteric fracture (S721), or Subtrochanteric fracture of femur (S722). Spine fracture were defined as Fracture of thoracic vertebra (S220) or Fracture of lumbar vertebra (S320).

### Statistical analyses

To analyze the hazard ratio (HR) for fracture (distal radius, hip, and spine fracture) in each severe or profound hearing loss group, the Cox proportional hazard model was used. In this analysis, crude (simple) and adjusted (age, sex, income, and region of residence) models were used. Bonferroni correction was used in this analysis to adjust the expected value of a wrongly declined null hypothesis. Two-tailed analyses were conducted, and P values less than 0.05 were considered to indicate significance. The results were statistically analyzed using SPSS v. 21.0 (IBM, Armonk, NY, USA).

## Results

The general characteristics of age, sex, income, and region of residence were exactly matched between the severe or profound hearing impairment and control groups (Table 1). The incidence of distal radius fracture, hip fracture, and spine fracture was 3.2% (154/4,854), 2.9% (140/4,854), and 6.9% (335/4,854) in the severe hearing-impaired group (S1 Table). In the profound hearing-impaired group, 2.4% (32/1,354), 2.2% (30/1,354), and 2.4% (32/1,354) showed distal radius fracture, hip fracture, and spine fracture.

**Table 1:** General characteristics of participants.

Characteristics		Severe hearing impairment (matched 1:4)			Profound hearing impairment (matched 1:4)		
		Hearing impairment	Control group	Rate (%)	Hearing impairment	Control group	Rate (%)
Age (years old)							
	0–4	16	64	0.3	27	108	2.0
	5–9	17	68	0.4	22	88	1.6
	10–14	27	108	0.6	30	120	2.2
	15–19	29	116	0.6	24	96	1.8
	20–24	41	164	0.8	47	188	3.5
	25–29	61	244	1.3	65	260	4.8
	30–34	96	384	2.0	64	256	4.7
	35–39	160	640	3.3	90	360	6.6
	40–44	267	1,068	5.5	127	508	9.4
	45–49	332	1,328	6.8	111	444	8.2
	50–54	399	1,596	8.2	107	428	7.9
	55–59	524	2,096	10.8	131	524	9.7
	60–64	700	2,800	14.4	140	560	10.3
	65–69	754	3,016	15.5	127	508	9.4
	70–74	667	2,668	13.7	117	468	8.6
	75–79	483	1,932	10.0	74	296	5.5
	80–84	208	832	4.3	36	144	2.7
	85+	73	292	1.5	15	60	1.1
Sex							
	Male	2,708	10,832	55.8	768	3,072	56.7
	Female	2,146	8,584	44.2	586	2,344	43.3
Income							
	1 (lowest)	384	1,536	7.9	272	1,088	20.1
	2	447	1,788	9.2	130	520	9.6
	3	306	1,224	6.3	88	352	6.5
	4	376	1,504	7.7	142	568	10.5
	5	365	1,460	7.5	100	400	7.4
	6	407	1,628	8.4	102	408	7.5
	7	378	1,512	7.8	84	336	6.2
	8	441	1,764	9.1	112	448	8.3
	9	471	1,884	9.7	91	364	6.7
	10	591	2,364	12.2	120	480	8.9
	11 (highest)	688	2,752	14.2	113	452	8.3
Region of residence							
	Urban	1,993	7,972	41.1	531	2,124	39.2
	Rural	2,861	11,444	58.9	823	3,292	60.8

The HR for distal radius fracture was higher in the severe hearing impairment group than the control group in both the crude and adjusted models (adjusted HR = 1.34, 95% confidence interval [95% CI] = 1.12–1.61, P < 0.001) (Table 2). The HR for hip fracture in this group was 1.67-fold higher than that in the control group after adjusting for confounders (95% CI = 1.38–2.03, P < 0.001), and the adjusted HR for spine fracture in this group was 1.44-fold higher than that in the control group (95% CI = 1.27–1.62, P < 0.001). The profound hearing impairment group demonstrated a higher HR for hip fracture than did the control group (adjusted HR = 2.21, 95% CI = 1.44–3.39, P < 0.001), and the adjusted HR for spine fracture in this group was 1.62-fold higher than that in the control group (95% CI = 1.25–2.09, P < 0.001).

**Table 2:** Crude and adjusted hazard ratios (95% confidence interval) of hearing impairment for distal radius/hip/thoracic and lumbar fracture.

Characteristics		Severe hearing impairment				Profound hearing impairment			
		Crude	P-value	Adjusted†	P-value	Crude	P-value	Adjusted†	P-value
Distal radius fracture			0.002*		0.001*		0.666		0.727
	Yes	1.32 (1.10–1.58)		1.34 (1.12–1.61)		0.92 (0.63–1.35)		0.93 (0.64–1.37)	
	No	1.00		1.00		1.00		1.00	
Hip fracture			<0.001*		<0.001*		0.008*		<0.001*
	Yes	1.49 (1.23–1.80)		1.67 (1.38–2.03)		1.78 (1.16–2.72)		2.21 (1.44–3.39)	
	No	1.00		1.00		1.00		1.00	
Thoracic and lumbar spine fracture			<0.001*		<0.001*		0.001*		<0.001*
	Yes	1.36 (1.20–1.53)		1.44 (1.27–1.62)		1.52 (1.17–1.96)		1.62 (1.25–2.09)	
	No	1.00		1.00		1.00		1.00	

* Cox-proportional hazard regression model, Significance at P < 0.05 with Bonferroni correction

† Adjusted model for age, sex, income, and region of residence

According to the age subgroups, in the < 60-year-old group, the HR for distal radius fracture and spine fracture in the severe hearing impairment group was higher than that the control group (adjusted HR = 1.52, 95% CI = 1.10–2.11, P = 0.011 for distal radius fracture; adjusted HR = 1.69, 95% CI = 1.22–2.34, P = 0.002 for spine fracture) (Table 3). The HR for spine fracture in the profound hearing impairment group was higher than that in control group (adjusted HR = 2.33, 95% CI = 1.40–3.89, P = 0.001). In the ≥ 60-year-old group, the incidence of hip and spine fractures was higher in the severe hearing impairment group than in the control group (adjusted HR = 1.70, 95% CI = 1.40–2.08, P > 0.001 for hip fracture; adjusted HR = 1.39, 95% CI = 1.22–1.58, P > 0.001 for spine fracture). Hip fracture was higher in the profound hearing impairment group than in the control group (adjusted HR = 2.11, 95% CI = 1.34–3.33, P = 0.001).

**Table 3:** Crude and adjusted hazard ratios (95% confidence interval) of hearing impairment for distal radius/hip/thoracic and lumbar fracture in subgroup analysis according to age.

Characteristics		Severe hearing impairment				Profound hearing impairment			
		Crude	P-value	Adjusted†	P-value	Crude	P-value	Adjusted†	P-value
		Age < 60 years old (n = 9,845)				Age < 60 years old (n = 4,225)			
Distal radius fracture			0.010*		0.011*		0.069		0.068
	Yes	1.53 (1.11–2.12)		1.52 (1.10–2.11)		0.57 (0.31–1.04)		0.57 (0.31–1.04)	
	No	1.00		1.00		1.00		1.00	
Hip fracture			0.882		0.866		0.115		0.109
	Yes	1.07 (0.44–2.63)		1.08 (0.44–2.65)		2.77 (0.78–9.81)		2.82 (0.79–9.98)	
	No	1.00		1.00		1.00		1.00	
Thoracic and lumbar spine fracture			0.002*		0.002*		0.001*		0.001*
	Yes	1.68 (1.21–2.32)		1.69 (1.22–2.34)		2.32 (1.39–3.86)		2.33 (1.40–3.89)	
	No	1.00		1.00		1.00		1.00	
		Age ≥ 60 years old (n = 14,425)				Age ≥ 60 years old (n = 2,545)			
Distal radius fracture			0.027		0.040		0.125		0.134
	Yes	1.28 (1.03–1.59)		1.26 (1.01–1.56)		1.49 (0.90–2.46)		1.47 (0.89–2.44)	
	No	1.00		1.00		1.00		1.00	
Hip fracture			<0.001*		<0.001*		0.004*		0.001*
	Yes	1.61 (1.33–1.96)		1.70 (1.40–2.08)		1.96 (1.25–3.09)		2.11 (1.34–3.33)	
	No	1.00		1.00		1.00		1.00	
Thoracic and lumbar spine fracture			<0.001*		<0.001*		0.015*		0.019
	Yes	1.38 (1.21–1.58)		1.39 (1.22–1.58)		1.45 (1.08–1.95)		1.43 (1.06–1.92)	
	No	1.00		1.00		1.00		1.00	

* Cox-proportional hazard regression model, Significance at P < 0.05 with Bonferroni correction

† Adjusted model for age, sex, income, and region of residence

## Discussion

The severe hearing impairment group demonstrated a higher risk of distal radius, hip, and spine fractures than the control group in the present study. The profound hearing impairment group also had a higher incidence of hip and spine fractures than did the control group. The older age of the severe hearing impairment group than profound hearing impairment group might result in high crude fracture rate and the risk of radius fracture in severe hearing impairment group. Approximately 59.4% and 37.6% were 60 or older in severe hearing impairment group and profound hearing impairment group, respectively. This increased fracture risk was present in both the < 60-year-old and ≥ 60-year-old subgroups. Few previous studies reported the risk of fracture in the hearing-impaired population according to age and fracture region.

Increased accidental traumatic injuries in hearing-impaired subjects could elevate fracture risk. Hearing-impaired subjects may be susceptible to falls due to decreased spatial orientation and speech perception [15]. To support this, previous studies reported the improved postural stability and gait after hearing aid or cochlear implant [16,17]. Although only severe falls might result in fractures, fracture could be used as a surrogate marker for risk of falls [18]. Additionally, deprivation of auditory perception decreased the daily activities of living, resulting in physical inactivity [15]. This low physical activity was associated with an increased risk of fracture in children [19].

Combined vestibular dysfunction could contribute to the increased risk of falls in hearing-impaired subjects [20]. The labyrinth is composed of the vestibular labyrinth as well as the cochlear labyrinth. The vestibular and cochlear labyrinths are connected in the otic capsule and have a common embryonic origin. Therefore, several inner ear diseases, such as Meniere’s disease, cause both auditory and vestibular dysfunction. Additionally, high-frequency hearing impairment was related to decreased cervical vestibular-evoked myogenic potential, which represents saccular dysfunction in elderly populations [20]. The saccule is an otolith organ involved in the detection of vertical linear movement and sensing of gravitational changes. The resultant decreased equilibrium in hearing-impaired subjects could increase the risk of falls and subsequent fractures.

Decreased cognitive function due to the elevated cognitive load in hearing-impaired subjects could elevate the risk of falls and fracture [21]. Equilibrium and gait require coordinated activity of several cognitive functions, including visuospatial abilities, executive-attentional function, and memory function [22]. Several previous studies reported that the decline in cognitive function elevated the risk of falls [22–24]. Hearing impairment influenced generalized cognitive function [25], and hearing loss was associated with dementia [26]. Although underlying pathophysiological mechanisms were not elucidated, several possible mechanisms were proposed. Both hearing loss and cognitive dysfunction may originate from general neural degeneration. Moreover, increased cognitive loading due to auditory perception deficits could result in cognitive depletion in hearing-impaired subjects.

Osteoporotic changes in the auditory system and in other bones, including the skeletal bones and spine, could result in fracture susceptibility in hearing-impaired subjects. Low cochlear capsule demineralization has been found to correlate with hearing loss in subjects with bone disorders such as osteogenesis imperfecta, Paget’s disease, and otosclerosis [27–29]. In addition, osteoporotic metabolic changes have been suggested in general hearing-impaired subjects, although the relationship between these changes and hearing impairment is still under debate. Several previous studies suggested that hearing impairment is related to decreased bone mineral density (BMD) in older adults (OR = 5.30, 95% CI = 1.20–23.26) [30]. However, there was no association between BMD and hearing impairment in Korean postmenopausal women [31]. The evaluation of BMD and the degree of hearing impairment were varied in previous studies, which may be a cause of the discordant results. In addition, the osteoporotic otic capsular changes could be smaller than other skeletal bony changes because the otic capsule consists of hard temporal bone. However, both the otic capsular bone and the cochlear hair cell changes in animal studies, such as the calcium channel expression in osteoporosis, could affect hearing impairment [32].

The present study used a representative large population across the lifespan. The validity of the sampling procedure was verified based on a previous study [13]. There were no missing participants because the NHIS data included all of the citizens of the nation without exception. In addition, control groups were randomly selected and matched according to age, sex, income, and region of residence. Because income and region of residence determine the availability of medical care, matching these variables was important, and the participants’ incomes were accurately collected based on the Korean NHIS data. The hearing impairment diagnosis was evaluated strictly based on a three-time testing of pure tone audiometry and an auditory brainstem response. Similarly, fracture diagnoses were determined using ICD-10 codes. However, some limitations should be considered when interpreting the present results. Several confounding variables associated with fractures were not adjusted in this study. The bone mineral density and T-score could not be considered in the present study. Hypertension, diabetes, depression, body mass index, level of physical activity, and smoking increased the risk of major osteoporotic fractures [33]. In addition, the determined duration of hearing impairment varied among the study population because the onset date of hearing impairment could not be obtained. The heterogeneous duration of hearing impairment could affect the relationship between hearing impairment and fracture risk.

## Conclusion

Severe hearing impairment increased the risk of distal radius, hip, and spine fractures across the lifespan. Both the < 60-year-old and ≥ 60-year-old subgroups had an increased risk of spine fractures compared with the control groups.

## Supporting information

S1 Table Fracture of each hearing impairment. (DOCX)
