# Supplementary material for: Hearing impairment increases the risk of distal radius, hip, and spine fractures: A longitudinal follow-up study using a national sample cohort
Source: PLoS One. 2018 Feb 13;13(2):e0192820. doi: 10.1371/journal.pone.0192820 (PMC5811044; doi:10.1371/journal.pone.0192820)
Supplement: S1 Table — (DOCX) [file pone.0192820.s001.docx]

**S1 Table** Fracture of each hearing impairment

| Characteristics | | Severe hearing impairment (matched 1:4) | | | Profound hearing impairment (matched 1:4) | | |
| --- | --- | --- | --- | --- | --- | --- | --- |
|  |  | Hearing impairment (n, %) | Control group (n, %) | P-value | Hearing impairment (n, %) | Control group (n, %) | P-value |
| Distal radius fracture | |  |  | 0.088 |  |  | 0.429 |
|  | Yes | 154 (3.2) | 507 (2.6) |  | 32 (2.4) | 149 (2.8) |  |
|  | No | 4,700 (96.8) | 18,908 (97.4) |  | 1322 (97.6) | 5,267 (97.2) |  |
| Hip fracture | |  |  | 0.002* |  |  | 0.023* |
|  | Yes | 140 (2.9) | 414 (2.1) |  | 30 (2.2) | 74 (1.4) |  |
|  | No | 4,714 (97.1) | 19,002 (97.9) |  | 1,324 (97.8) | 5,342 (98.6) |  |
| Thoracic and lumber spine fractrue | |  |  | 0.001* |  |  | 0.005* |
|  | Yes | 335 (6.9) | 1,086 (5.6) |  | 32 (2.4) | 149 (2.8) |  |
|  | No | 4,519 (93.1) | 18,330 (94.4) |  | 1,322 (97.6) | 5,267 (97.2) |  |

Chi-square test, Significant at P < 0.05
